# Silicon builds resilience in strawberry plants against both strawberry powdery mildew *Podosphaera aphanis* and two-spotted spider mites *Tetranychus urticae*

**DOI:** 10.1371/journal.pone.0241151

**Published:** 2020-12-08

**Authors:** Bo Liu, Keith Davies, Avice Hall

**Affiliations:** 1 Department of Clinical, Pharmaceutical and Biological Sciences, School of Life and Medical Sciences, University of Hertfordshire, Hatfield, Hertfordshire, United Kingdom; 2 Division of Biotechnology and Plant Health, Norwegian Institute of Bioeconomy Research, Ås, Norway; Banaras Hindu University, INDIA

## Abstract

Silicon is found in all plants and the accumulation of silicon can improve plant tolerance to biotic stress. Strawberry powdery mildew (*Podosphaera aphanis*) and two-spotted spider mite (*Tetranychus urticae*) are both detrimental to strawberry production worldwide. Two field trials were done on a UK commercial strawberry farm in 2014 and 2015, to assess the effects of silicon nutrient applied via the fertigation system on *P*. *aphanis* and *T*. *urticae*. The silicon treatments decreased the severity of both *P*. *aphanis* and *T*. *urticae* in two consecutive years on different cultivars. The percentage leaf area infected with *P*. *aphanis* mycelium from silicon treated plants were 2.19 (in 2014) and 0.41 (in 2015) compared with 3.08 (in 2014) and 0.57 (in 2015) from the untreated plants. The etiology of the pathogen as measured by the Area Under the Disease Progress Curve from silicon (with and without fungicides) treatments was 152.7 compared with 217.5 from non-silicon (with and without fungicides) treatments for the overall period of 2014–2015. The average numbers of *T*. *urticae* recorded on strawberry leaves were 1.43 (in 2014) and 1.83 (in 2015) in plants treated with silicon compared with 8.82 (in 2014) and 6.69 (in 2015) in untreated plants. The silicon contents of the leaves from the silicon alone treatment were 26.8 μg mg^-1^ (in 2014) and 22.2 μg mg^-1^ (in 2015) compared with 19.7 μg mg^-1^ (in 2014) and 21.4 μg mg^-1^ (in 2015) from the untreated. The silicon nutrient root application contributed to improved plant resilience against *P*. *aphanis* and *T*. *urticae*. Silicon could play an important role in broad spectrum control of pests and diseases in commercial strawberry production.

## Introduction

Silicon (Si) is the second most abundant mineral element in the soil and constitutes ca. 28% of the earth’s crust [1, 2]. Silicon is found in all plants but not considered an essential element for plant growth (International Plant Nutrition Institute, http://www.ipni.net/nutrifacts-northamerican), as it is not directly involved in the plant metabolic process [2]. Nevertheless, the beneficial effects of silicon have been observed in many dicotyledon and monocotyledon species [3]. Nowadays, silicon is referred to as “quasi-essential” for the growth of higher plants due to its important role in alleviating biotic and abiotic stresses [4]. A bioavailable form of silicon (H_4_SiO_4_) is taken up by plants when the soil solution pH is < 9, and it is transported through the xylem and deposited in the leaf epidermal cells and cell walls of many higher plants as a silica gel (a form of hydrated amorphous silica, SiO_2_ • nH_2_O, or polymerized silicic acid) [4, 5].

Silicon accumulation in plants improves their tolerance, especially when they are under biotic or abiotic stress [6]. For example, silicon reduces severity of epidemics of rice brown spot [7], melon [8], cucumber and barley powdery mildews [3, 9]. Silicon has also been found to suppress insect pests such as rice leaf folder (*Cnaphalocrocis medinalis*), sugarcane borer (*Diatraea saccharalis*) and pyralid borer (*Eldana saccharina*) [3]. Silicon application not only reduced powdery mildew severity on courgettes by 35% but also improved the efficacy of biocontrol agents that were applied on courgette leaves [10]. Silicon is used routinely by growers as a surfactant/wetter in fungicide applications to improve the spray coverage of leaves [11, 12]. In strawberry, the use of silicon surfactants increased the efficacy of acaricide products for controlling tarsonemid mites (*Phytonemus pallidus* spp. *fragariae* (Zimmerman)) [13]. Silicon in grasses can act as a deterrent to chewing insects and small mammalian herbivores through the presence of silica phytoliths, which provide a resistance barrier to feeding invertebrates [3].

These effects can be explained by the deposition of silicon in leaves, stems and hulls [2, 14]. For example, silicon deposited under the cuticle acts as a cuticle-silicon double barrier to reduce transpiration and prevent penetration by fungi and insects [4, 15]. Previous work demonstrated that strawberry treated with a silicon nutrient had increased leaf wax density and thicker leaf cuticles, which correlated with reduced susceptibility to strawberry powdery mildew [15]. Silicon was also found to enhance trichome growth on adaxial and abaxial surfaces of strawberry leaves [16]. Furthermore, soluble silicon acts as a regulator of host resistance to pathogens through the interaction with plant stress signalling systems and the stimulation of plant defence compounds [17].

Growers in the UK have increasingly become aware of strawberry being susceptible to both powdery mildew and the two-spotted spider mite. Strawberry powdery mildew, caused by *Podosphaera aphanis* (Wallr.), is a major fungal disease affecting strawberry production worldwide [18]. The pathogen is characterized as an obligate biotroph [19]. Serious epidemics can reduce crop yields as a result of inadequately ripened fruits, fruit deformation, poor flavour development and reduced storage life. The pathogen infects strawberries on nearly all plant parts including leaves, flowers, fruits, pedicels and peduncles, and is specific to this crop [18]. Temperature, relative humidity (RH), light intensity, cultivar and leaf phenology were found to affect *P*. *aphanis* conidial germination, germ tube elongation, conidiation and disease severity [20]. Studies suggested that the optimal environmental conditions for conidial germination were 15–30°C with RH >60% [18]. Growers routinely use fungicides to prevent or control powdery mildew infection. It has been shown that the best time to start to control *P*. *aphanis* is before conidia or before ascospores are released, to avoid rapid spread of the pathogen [18].

The two-spotted spider mite *Tetranychus urticae* (Koch.) is a major global pest of crops grown in field and glasshouse conditions [21]. The mites feed on leaf cell contents using their piercing-sucking mouthparts [22], causing leaf bronzing; they also produce webs on the leaf surface, which reduce the leaf photosynthetic ability and result in reduced crop yield and quality [23]. Strawberry fruits from mite infested plants had an increased level of acidity and decreased levels of anthocyanin and phenolic compounds compared to those from healthy plants, suggesting poorer fruit quality [23]. Two-spotted spider mites have become a serious threat to horticulture growers, partly because the large scale use of chemical insecticides has induced their insensitivity to chemical acaricides (e.g. cyhexatin, dicofol and azocyclotin) [24]. The intensive use of such insecticides has also reduced populations of their natural enemies, enabling the mite populations to increase exponentially [25].

As a result of EU directives to reduce the application of synthetic chemicals for the control of pests and diseases, alternative crop protection management strategies are of increasing importance to growers. Therefore, the aim of this investigation was to explore the effects of the silicon nutrient delivered via the fertigation system on strawberry powdery mildew *P*. *aphanis* and the mite *T*. *urticae* in a commercial cropping situation.

## Materials and methods

### Description of the experimental site

Silicon fertigation field experiments were done in 2014 and 2015 on a commercial strawberry farm at Wisbech, Cambridgeshire, UK (PE14 0HS). The farm has a total cropping area of 113 ha, within which 14 ha was used for growing strawberries. The use of the commercial farm ensured that strawberries were being grown in optimum conditions to meet supermarket standards. Both field trials were set up in polyethylene tunnels commercially managed and harvested; such a commercial design enabled the results to be applicable for commercial growers. Each tunnel had five raised soil beds (180m long) running in parallel (1m spacing between beds) (S1A Fig). Six strawberry plants were grown in each coir bag (1m long) placed on raised soil beds (S1B Fig). There were approximately 5,000–5,300 strawberry plants in one tunnel. Water (from the mains) and nutrients were delivered to the plants through irrigation drippers (four per coir bag) connected to the fertigation system (an irrigation system that delivers both water and fertilizers) five times per day, and commercial fungicide applications were done based on the normal farm spray schedule. The grower also used biocontrol agents (e.g. *Beauveria bassiana*) to control strawberry pests such as two-spotted spider mites. The silicon product used was Sirius^®^ (a nutrient to increase the strength and health of the plants, main active ingredient: 70–80% tetraethyl silicate) provided by Orion Future Technology (Kent, UK). The silicon nutrient was added in the irrigation water at a concentration of 0.017% (by volume, 0.003 mg ml^-1^ in the irrigation water) and applied once per week via the fertigation system. The adoption of the farm fertigation system for silicon treatments necessitated the use of large sample sizes (e.g. 15 leaves x five replicates per treatment), because feeding through the irrigation pipes could be controlled only on a tunnel basis. The leaf assessments of severity of powdery mildew and numbers of spider mites were done every two weeks.

### 2014–2015 silicon fertigation experiments

The experiment in 2014 was set up in Blackberry Field in April (S2A Fig). Plants of strawberry cultivar ‘Driscoll Jubilee^TM^’ (June bearer—one harvest per year in June/July) [26] were planted in coir bags on 20 March 2014. Plants were then covered by fleece to protect them from frost until late April. Application of the silicon nutrient started on 09 May and four treatments were used in two tunnels between 09 May and 12 August 2014.

In one tunnel, no silicon was applied. The first 15m of five growing beds received no fungicide sprays (untreated control) and the remaining parts of beds received fungicide sprays in accordance with commercial spraying practice (commercial fungicide only) (Table 1). In another nearby (< 10m distance) tunnel, all five beds received 0.017% silicon nutrient through the fertigation system once per week. The first 15m of each bed received no fungicide (0.017% Si alone) and the remaining parts of beds received commercial fungicide according to the normal farm practice (0.017% Si plus commercial fungicide).

**Table 1:** Fungicide/biocontrol agent applications following the farm commercial spray schedules in the 2014 and 2015 silicon fertigation experiments.

Date of fungicide application in 2014 or 2015	2014 Blackberry Field experiment^a^	2015 Pheasant Field experiment^a^
10 April	NA^b^	Boscalid & Pyraclostrobin, Quinoxyfen
24 April	NA	Cyprodinil & Fludioxonil, Myclobutanil
04 May	NA	Cyprodinil & Fludioxonil, Myclobutanil
09 May	Fenhexamid, Bupirimate	NA
16 May	Fenhexamid	NA
29 May	Azoxystrobin	NA
09 Jun	Fenhexamid	NA
18 Jun	Fenhexamid	NA
27 Jun	*Beauveria bassiana*^c^	NA
04 Jul	Fenhexamid	NA
06 Jul	NA	Bupirimate, Fenhexamid
08 Jul	*Beauveria bassiana*	NA
14 Jul	NA	Sulphur
31 Jul	NA	Myclobutanil, Pyrimethanil
08 Aug	Sulphur	NA
14 Aug	NA	Azoxystrobin, Boscalid & Pyraclostrobin
15 Aug	Azoxystrobin	NA
20 Aug	NA	Fenhexamid
27 Aug	NA	Fenhexamid
29 Aug	Sulphur	NA
11 Sep	NA	Azoxystrobin
12 Sep	Pyrimethanil, Bupirimate	NA

Names of fungicide active ingredients are provided in the table. Silicon nutrient was applied to plants through the fertigation tubes once per week starting on 09 May in 2014 and on 22 April in 2015.

^a^The 2014 and 2015 experiments consisted of four treatments, each treatment consisted of five growing beds (i.e. five replicates, each 15m long) running in parallel, which were: 1) a block of five untreated control beds, 2) a block of commercial fungicide treated beds, 3) a block treated with commercial fungicide and 0.017% Si nutrient (by volume) applied weekly and 4) a block treated with only 0.017% Si nutrient at weekly intervals (i.e. no fungicide treatments).

^b^ Not applicable, no fungicide application was made.

^c^A biocontrol agent used by the grower against strawberry pests (e.g. aphids, whiteflies etc.).

The experiment in 2015 was set up in Pheasant Field in April (S2B Fig). Plants of strawberry cultivar ‘Driscoll Amesti^TM^’ (Everbearer–harvesting from July to October) [26] were planted in coir bags on raised beds on 05 March 2015. Plants were then covered by fleece until 08 April. Application of the silicon nutrient started on 22 April 2015 and the same four treatments as in 2014 were used in two nearby (< 10m apart) tunnels between 22 April and 29 September 2015.

### Assessment of *Podosphaera aphanis* development on the leaf surface

The assessment of *P*. *aphanis* development was based on 75 leaves per treatment (five replicates of 15 leaves per strawberry bed). Pre-assessments before the silicon treatment started on 08 and 22 April in 2014 and on 21 April in 2015, respectively. Samples were collected at two-weekly intervals commencing 08 April 2014 and 21 April 2015. Each leaflet of the sampled leaf was placed under a dissecting microscope (GX microscopes, 1x and 3x objectives; 10x eyepieces, GT Vision Ltd, Suffolk, UK) to be assessed for *P*. *aphanis* development using the assessment key developed by Jin [15]. The disease severity was expressed as % leaf area covered by *P*. *aphanis* colonies (amount of mycelium).

### Assessment of *Tetranychus urticae* presence on the leaf surface

The same batches of leaf samples collected for *P*. *aphanis* assessment (20 May-12 August in 2014 and 21 April-11 August in 2015) were also used for the assessment of *T*. *urticae* infestation. Each sampled leaf was placed under a dissecting microscope (as described above for *P*. *aphanis* assessment) at ×10 magnification, and the number of *T*. *urticae* (at all life-stages apart from eggs) that were present on the leaf surface was recorded. The observation of the leaf was done by moving the leaf from the right to the left side in a regular pattern and observations started from the top part of the leaf surface then moved systematically to the bottom part of the leaf ensuring that every part of the leaf surface was observed without repetition, thereby avoiding miscounting or double counting. In addition, if the movement of *T*. *urticae* followed the same route as the observation, its movement direction was specially noted, and it was excluded from the count if it appeared in the next observation field. Since the strawberry leaf was observed under x10 magnification, one observation field could cover a relatively large percentage of the leaf area (25% of the whole leaf in most cases), thus avoiding multiple counting due to spending a long time on the same leaf. Also, since the size of the *T*. *urticae* was small compared with the observation field, its movement usually stayed within the same field. Even if it was moving, its movement direction could easily be traced; therefore the risk of repeated counting could be minimized.

### The analysis of 2014 and 2015 experimental results

The analysis was done for the 2014 and 2015 seasons individually, as well as for the combined seasons to assess the consistency of the effects of silicon on *P*. *aphanis* development and *T*. *urticae* presence.

Area Under the Disease Progress Curve (AUDPC) (The American Phytopathological Society, https://www.apsnet.org) was used for the analysis of *P*. *aphanis* development. The calculation was done using the equation AUDPC = $\sum_{i=1}^{n-1}((x_{i+1}+x_{i})/2)(t_{i+1}-t_{i})$, where x_i_ is a measure of disease severity (% area of leaf coverage by *P*. *aphanis*) at the i^th^ sampling date, t is a measure of time (i.e. days), and n is the total number of samplings. The calculation of the AUDPC for each treatment was based on fortnightly disease assessment results from five strawberry beds. Each bed was a replicate, which had an AUDPC value based on the average % area of leaf coverage by *P*. *aphanis* mycelium of 15 leaves collected randomly from this bed. Comparisons of five AUDPC values for each of those four treatments were made by the analysis of variance (ANOVA) test using R software (The R Foundation for Statistical Computing, 3.3.0 GUI 1.68). In addition, apparent infection rate (r) was calculated to assess the rate of epidemic development for each treatment using the equation r = (lnq_2_- lnq_1_)/(t_2_-t_1_), where q_2_ and q_1_ are the quantities of disease present at times t_1_ and t_2_, respectively [27].

Similar to the calculation of the AUDPC, the overall sum of the number of *T*. *urticae* for the entire experimental period could be represented by the overall Area Under the ‘Pest’ Progress Curve (AUPPC). The AUPPC value for each sampling date was calculated based on the mean value of the total number of *T*. *urticae* on 15 leaf samples from each strawberry bed of all five beds (each bed was a replicate, five replicates in total). The overall analysis for the combined two seasons was based on the overall number of spider mites per treatment in 2014 and 2015 and five sum values from each treatment were compared using ANOVA.

### Silicon extraction

Three mature strawberry leaves were collected (one leaf per plant) per strawberry bed for silicon extraction. Three beds from each treatment were sampled (i.e. three replicates of 3 leaves per treatment). The Autoclave-induced digestion (AID) [28] method was used to extract silicon from strawberry leaves (oven dried (at 60°C) leaf powder (0.1g) per three leaf samples x three replicates per treatment). The leaf silicon extraction was done monthly between 08 April and 23 September in 2014 and between 21 April and 29 September in 2015. A silicon standard curve was made to calculate the concentration of silicon in the strawberry plant material [15]. The silicon concentration of the sample material was then calculated by using the equation y = 1.0957x, where y is the absorbance of silicon at 650nm (CECIL 1021 Spectrophotometer, 1000 series, Cambridge, UK) and x is the concentration of silicon (mg ml^-1^).

## Results

Results from both 2014 and 2015 experiments were consistent; strawberry plants that received weekly silicon application (with or without fungicide) had reduced severities of both *P*. *aphanis* and *T*. *urticae* compared with the untreated control plants.

### Assessment of *Podosphaera aphanis* development on the leaf surface

**Fig 1:**
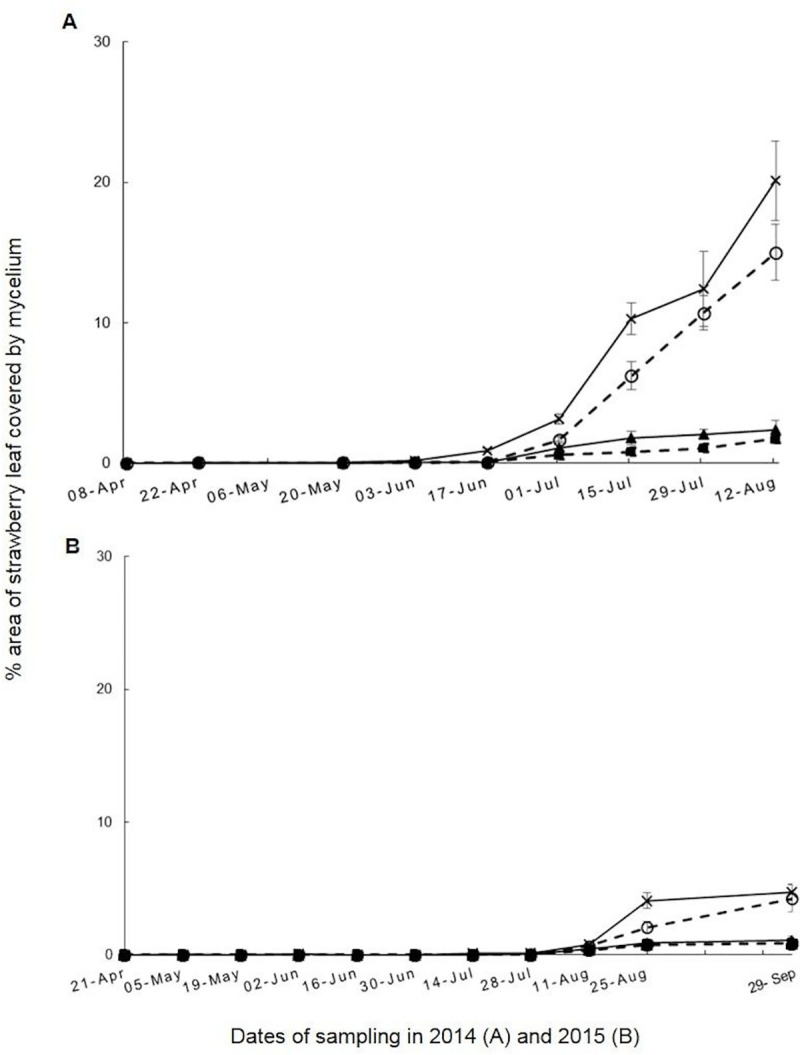
Percentage strawberry leaf area covered by *Podosphaera aphanis* mycelium plotted against time in 2014–2015 experiments. Treatments in (A) Blackberry Field in 2014 (08 April-12 August) and in (B) Pheasant Field in 2015 (21 April-29 September) were: untreated control 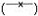, commercial fungicide only 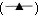, 0.017% silicon nutrient (by volume) alone applied once a week without commercial fungicide) 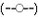, 0.017% silicon nutrient (by volume) applied once a week, plus commercial fungicide 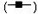. Vertical axis indicates mean % area of strawberry leaf covered by mycelium (75 leaves per treatment). Horizontal axis shows dates of sampling with a total of 9 (in 2014) and 11 (in 2015) samplings during the experimental period. AUDPC and infection rate *r* values were calculated. Error bars represent standard errors of means of five replicates.

In the 2014 experiment, strawberry plants from the 0.017% Si plus commercial fungicide treatment had the smallest disease scores (AUDPC = 63) (*P* < 0.001) and infection rate (r = 0.0012) among all treatments (Table 2; Fig 1A). There was a significant difference (*P* < 0.05) in disease severity between the untreated control (AUDPC = 662) and 0.017% Si alone (AUDPC = 475) treatments from 17 June 2014 onwards. In addition, it was shown that the onset of epidemic development was delayed by approximately 14 days for two silicon treatments (17 June 2014) compared with the untreated control (03 June 2014) (Fig 1A). Furthermore, the 0.017% Si plus commercial fungicide treatment had a smaller disease severity compared with commercial fungicide only treatment (AUDPC = 106, r = 0.0017) (Table 2), which indicated that the use of silicon and fungicide together may enhance the effectiveness of fungicide treatments.

**Table 2:** The analysis of the severity of *Podosphaera aphanis* (AUDPC^a^ & r (Apparent infection rate)^b^) and *Tetranychus urticae* (AUPPC^c^) for the treatments in 2014 and 2015 experiments.

Treatment	2014 Blackberry Field experiment			2015 Pheasant Field experiment		
	AUDPC	*r*	AUPPC	AUDPC	*r*	AUPPC
**Untreated control**	662	0.0042	6,551	281	0.0011	13,149
**Commercial fungicide only**	106	0.0017	19,130	69	0.0005	8,149
**0.017% Si**^**d**^ **alone**	475	0.0036	2,222	267	0.001	2,265
**0.017% Si plus commercial fungicide**	63	0.0012	1,977	53	0.0004	2,681

**Fig 2:**
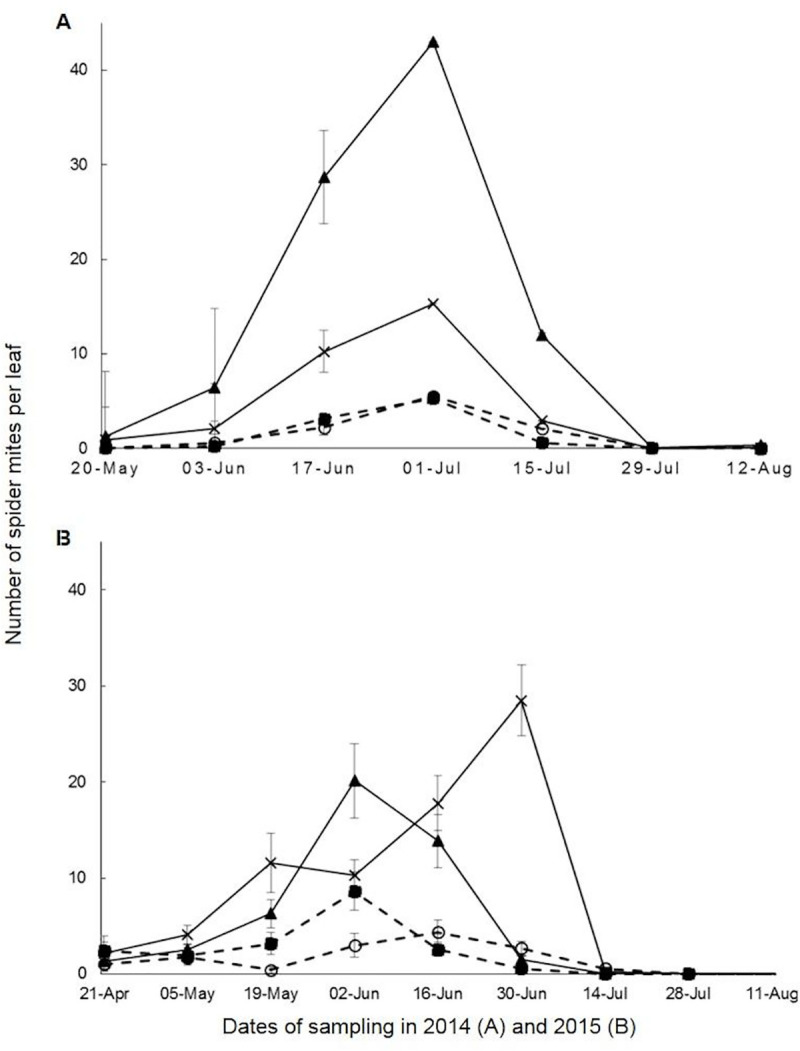
Numbers of *Tetranychus urticae* per strawberry leaf plotted against time in 2014–2015 experiments. Treatments in (A) Blackberry Field in 2014 (20 May–12 August) and in (B) Pheasant Field in 2015 (21 April–11 August) were: untreated control 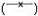, commercial fungicide only 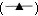, 0.017% silicon nutrient (by volume) alone applied once a week without commercial fungicide) 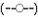, 0.017% silicon nutrient (by volume) applied once a week, plus commercial fungicide 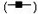. Vertical axis indicates average number of *T*. *urticae* counted per strawberry leaf of 75 leaves sampled from each treatment. Horizontal axis shows dates of sampling with a total of 7 (in 2014) and 9 (in 2015) samplings during the experimental period. AUPPC values refer to Table 2. Error bars represent standard errors of means of five replicates.

The two-weekly leaf assessment results are presented separately for *P*. *aphanis* (Fig 1) and *T*. *urticae* (Fig 2).

^a^The calculation was based on the two-weekly assessment of the average % area of strawberry leaf covered by *P*. *aphanis* mycelium (five replicates of 15 leaves each) in 2014 (08 April-12 August) and in 2015 (21 April-29 September).

^b^r refers to Apparent infection rate, the value indicates the rate of epidemic development [27].

^c^The value indicates the overall sum of *T*. *urticae* per treatment (five replicates of 15 leaves each) in 2014 (20 May-12 August) and in 2015 (21 April-11 August).

^d^Silicon nutrient was applied once per week at a concentration of 0.017% (by volume) in the irrigation water from 09 May in 2014 and from 22 April in 2015.

In the 2015 experiment, plants from 0.017% Si plus commercial fungicide treatment had the smallest disease severity (AUDPC = 53, r = 0.0004) throughout the experimental period (Table 2; Fig 1B). A significant difference (*P* < 0.001) in disease severity was found between this treatment and the untreated control (AUDPC = 281, r = 0.0011). Plants from this treatment developed less *P*. *aphanis* than those from the commercial fungicide only treatment (AUDPC = 69, r = 0.0005) (Table 2). Moreover, plants from the treatment 0.017% Si alone also developed less disease (AUDPC = 267, r = 0.001) than the untreated control (Fig 1B), which was consistent with the 2014 results (Fig 1A).

### Assessment of *Tetranychus urticae* presence on the leaf surface

Results from the 2014 experiment showed that 0.017% Si alone and 0.017% Si plus commercial fungicide treatments had average smaller numbers of *T*. *urticae* per strawberry leaf (< 2) than the untreated control and commercial fungicide only treatment (Fig 2A). The fungicide only treatment had an average of ten *T*. *urticae* per leaf, which was the highest among all treatments. The treatment 0.017% Si plus commercial fungicide had the smallest AUPPC value (AUPPC = 1,977) compared with the untreated control (AUPPC = 6,551), commercial fungicide only (AUPPC = 19,130) and 0.017% Si alone (AUPPC = 2,222) treatments (Table 2). It can be seen that both treatments with silicon had smaller values of AUPPC than treatments without silicon.

In addition, all four treatments showed a similar trend, with the number of *T*. *urticae* per leaf increasing from < 2 on 20 May 2014 to a maximum (> 40 in the commercial fungicide only treatment) on 01 July and then gradually decreasing to nearly 0 by late July/early August 2014 (Fig 2A). There was a significant difference between commercial fungicide only and 0.017% Si alone (*P* < 0.001) treatments, and between commercial fungicide only and 0.017% Si plus commercial fungicide (*P* < 0.001) treatments on 01 July 2014.

Results from the 2015 experiment showed that there were fewer *T*. *urticae* present on leaves from the silicon treatments than treatments without silicon throughout the experimental period (Fig 2B). Leaves from 0.017% Si alone and 0.017% Si plus commercial fungicide treatments had less *T*. *urticae* present. These two treatments had an average of two *T*. *urticae* per leaf compared to nine in the untreated control. Similarly, the 0.017% Si alone treatment had the smallest AUPPC value (AUPPC = 2,265), followed by the 0.017% Si plus commercial fungicide treatment (AUPPC = 2,681) (Table 2). The untreated control had a greater AUPPC value (AUPPC = 13,149) than the commercial fungicide only treatment (AUPPC = 8,149), which was slightly different from the 2014 results. The AUPPC results from 2014/2015 showed that treatments with silicon had less *T*. *urticae* infestation than the untreated control and commercial fungicide only treatments. Interestingly, for treatments with the same application rate of silicon, those with added fungicide had greater *T*. *urticae* infestation numbers than those without fungicide (i.e. commercial fungicide only vs. untreated control in 2014; 0.017% silicon nutrient plus commercial fungicide vs. 0.017% silicon nutrient in 2015) (Table 2).

### Overall analysis of combined 2014/2015 *Podosphaera aphanis* severity and *Tetranychus urticae* infestation results

Apart from the individual analyses of the 2014 and 2015 results, an overall analysis of all data from these two years was also done (Table 3). Strawberry plants that received silicon developed significantly less *P*. *aphanis* (AUDPC = 152.7) (*P* < 0.05) and were less infested by *T*. *urticae* (AUPPC = 170) (*P* < 0.001) compared with plants from non-silicon treatments (AUDPC = 217.5, AUPPC = 876) in both 2014 and 2015. It was shown that both strawberry powdery mildew severity (Fig 1) and numbers of spider mites present (Fig 2) were significantly less in the two silicon treatments (0.017% Si alone; 0.017% Si plus commercial fungicide) compared with the two non-silicon treatments (untreated control; commercial fungicide only) and this significant difference was consistent throughout 2014 and 2015 (Table 3).

**Table 3:** Overall analysis of strawberry powdery mildew symptoms and two-spotted spider mites infestation results in 2014 and in 2015.

	Percentage of strawberry leaf area covered by *P*. *aphanis* mycelium^b^			Number of *T*. *urticae* per strawberry leaf^b^		
Silicon fertigation experiment^a^	Treatments without silicon	Silicon treatments	*P*-value between treatments with and without silicon	Treatments without silicon	Silicon treatments	*P*-value between treatments with and without silicon
**2014 Blackberry Field**	3.08 ± 0.6	2.19 ± 0.4	0.0301	8.82 ± 1.1	1.43 ± 0.3	1.38e-07
**(08 April-12 August)**						
**2015 Pheasant Field**	0.57 ± 0.3	0.41 ± 0.2	0.0125	6.69 ± 2	1.83 ± 0.5	0.0004
**(21 April–29 September)**						
	AUDPC value^c^			AUPPC value^c^		
**Overall period 2014–2015**	217.5	152.7	0.0097^d^	876	170	1.28e-09^d^

Percentage leaf area covered by *Podosphaera aphanis* mycelium and numbers of *Tetranychus urticae* per leaf were assessed. The overall analysis was done by comparing the assessment results between two silicon treatments (0.017% Si alone and 0.017% Si plus commercial fungicide) and two treatments without silicon (untreated control and commercial fungicide only) in 2014 and in 2015.

^a^Silicon nutrient was applied once per week at a concentration of 0.017% (by volume) in the irrigation water via the fertigation system from 09 May in 2014 and from 22 April in 2015.

^b^Data are the mean value of five replicates ± standard error (mean ± SE).

^c^Data are the mean AUDPC/AUPPC values of five replicates at the final assessment from both 2014 and 2015, respectively.

^d^*P*-values for the overall period of 2014–2015 were calculated based on AUDPC (for *P*. *aphanis*) or AUPPC (for *T*. *urticae*) values of five replicates (i.e. five strawberry beds) from both 2014 and 2015 results using ANOVA.

There was a clear difference (*P* < 0.001) in powdery mildew severity between the 2014 and 2015 experiments. The 2014 experiment had much more severe disease (AUDPC = 662, r = 0.0042 in untreated control) than the 2015 experiment (AUDPC = 281, r = 0.0011 in untreated control) (Table 2; Fig 1). Nevertheless, when analysing the 2014 and 2015 disease results separately, even though disease severity differed between these two years, plants in the silicon treatments still had less powdery mildew than those in the non-silicon treatments (*P* < 0.05) in both years (Table 3). The disease AUDPC from the untreated control treatment was significantly higher (*P* < 0.05) in 2015 but only slightly higher (*P* < 0.1) in 2014 than that from the 0.017% Si alone treatment in the same year (Table 2). No significant difference was found between commercial fungicide only and 0.017% Si plus commercial fungicide treatments.

The numbers of *T*. *urticae* present were different between 2014 and 2015, especially in the untreated control (AUPPC = 6,551 in 2014, and 13,149 in 2015) and commercial fungicide only treatments (AUPPC = 19,130 in 2014, and 8,149 in 2015) (Table 2). Moreover, in the 2014 experiment, strawberry plants in the 0.017% Si alone treatment were less infested by *T*. *urticae* (AUPPC = 2,222) (*P* < 0.05) than those in the untreated control. Similarly, strawberry plants in the 0.017% Si plus commercial fungicide treatment were less infested by *T*. *urticae* (AUPPC = 1,977) (*P* < 0.001) than those which received only commercial fungicide. In the 2015 experiment, even though the overall numbers of *T*. *urticae* present were different from the previous year, strawberry leaves in the 0.017% Si alone treatment were observed to have fewer *T*. *urticae* (AUPPC = 2,265) than those in the untreated control (*P* < 0.05) (Fig 2B).

### Silicon content in strawberry plants in the 2014 and 2015 experiments

The leaf silicon content from the untreated control was significantly less (19.7 μg mg^-1^) than that in the 0.017% Si alone (26.8 μg mg^-1^) and 0.017% Si plus commercial fungicide (26.8 μg mg^-1^) treatments in 2014 (*P* < 0.001); however in 2015, there was a difference (*P* < 0.05) between the commercial fungicide only (21.1 μg mg^-1^) and the 0.017% Si plus commercial fungicide (25.3 μg mg^-1^) treatments (Table 4). In both years, there was a significant difference (*P* < 0.001) in amounts of leaf silicon content between the first assessment (19 April in 2014; 21 April in 2015) and following assessments in the same year.

**Table 4:** Mean monthly leaf silicon content (μg mg^-1^) from each treatment in 2014 (08 April-23 September) and 2015 (21 April-29 September) experiments.

Treatments	Mean monthly leaf silicon content tested^a^ (μg mg^-1^)	
	2014 experiment	2015 experiment
Untreated control	19.7^a^^b^ ± 1.3	21.4^a^^b^ ± 1.9
Commercial fungicide	23.7^a^^b^ ± 1.4	21.1^a^ ± 1.2
0.017% Si^c^	26.8^b^ ± 1.9	22.2^a^^b^ ± 1
0.017% Si plus commercial fungicide	26.8^b^ ± 1.8	25.3^b^ ± 1.4

^a^Data are the mean of three replicates ± standard error (mean ± SE).

^b^Data followed by same letter within each column indicate no significant difference (*P* > 0.05) between treatments using ANOVA followed by TukeyHSD test.

^c^Silicon nutrient was applied once per week at a concentration of 0.017% (by volume) in the irrigation water from 09 May in 2014 and from 22 April in 2015.

## Discussion

The work reported here has shown that strawberry plants treated with silicon were more resilient and had significantly less severe *P*. *aphanis* infection and less severe *T*. *urticae* infestation compared with those from the untreated control. This effect was found to be consistent over the two-year research and on different cultivars. Silicon has been used for disease control in many economic crops including barley, cucumber, rice and strawberry [29]. Studies showed that plants that received silicon were more resilient under the stresses of fungal diseases such as powdery mildew, rust, and leaf spot [30]. One explanation for this is that the accumulation of silicon in the form of amorphous silica, forms a barrier to prevent penetration by the pathogen [31]. Work has shown that silicon treatment of strawberry plants increased leaf cuticle thickness, density of leaf wax and numbers of leaf trichomes [15], and silicon treated coffee seedlings developed a thicker epicuticular wax layer [29], suggesting that silicon induces the formation of physical defence barriers against attack by pathogens and pests [32]. It was found that soluble silicon was effective as a preventive measure for increasing plant resistance at an early stage of pathogen colonisation (e.g. conidial germination, germ tube elongation etc.), and the effect was greater in very susceptible cultivars (up to 86%) compared to less susceptible cultivars (up to 58%) [33].

Silicon plays a role in inducing plant natural defence responses, by interacting with a number of key components of plant stress signalling systems [30]. For example, soluble silicon treated cucumber and rice demonstrated increased accumulation of phenolics and phytoalexins when infected by powdery mildew and blast [4, 30, 34]. Research on wheat also found that silicon reduces the production of reactive oxygen species (ROS), which causes oxidative damage to plant cells, therefore reducing the cellular damage caused by wheat blast (*Pyricularia oryzae*) [31]. Results from the present study showed that the rate of *P*. *aphanis* epidemic development in the 0.017% Si alone treatment was ca.14% less than that in the untreated control in the 2014 experiment; whereas it was only ca.9% less when compared with the untreated control in 2015, when there was a less disease severity than in 2014 (*P* < 0.05). This further indicated that plants may benefit more from the supply of silicon when disease severity is greater.

The field experiment results showed that treatment 0.017% Si plus commercial fungicide had the smallest disease severity in both 2014 and 2015. Recent study also found that strawberry plants received silicon with potassium carbonate were more resilient against *p*. *aphanis* (AUDPC = 6) compared with potassium carbonate treatment alone (AUDPC = 24) [11, 15]. The combined treatment also had a delay in onset of epidemic development of *P*. *aphanis*. Similar findings were also shown between treatments silicon with fungicides and fungicides alone in the same study [15]. The above evidence indicated that plants more benefit more from the combination of silicon and fungicides than the use of fungicides alone.

There was a different infestation level of *T*. *urticae* between 2014 and 2015 for the same treatment. The average number of *T*. *urticae* per leaf was two to three times greater in the untreated control than in the silicon treatments in 2014 and more than three times greater in 2015. This suggested that there could be an interaction between abiotic effects (e.g. temperature and humidity) and cultivars. Nevertheless, strawberry plants from 0.017% Si alone treatment still had fewer *T*. *urticae* compared with those from the untreated control over two years. Silicon has been reported to improve plants resistance against many pests such as spittlebug in sugarcane, rice green leafhopper, stem borer and brown planthopper [4, 35, 36]. Silicon can affect insect biology such as food intake, nymph development and adult longevity [37]. High concentration of silicon on sugarcane has been observed to extend the nymphal stage of spittlebug *Mahanarva fimbriolata* Stal and shorten the adult life [35]. Soluble silicon is particularly effective in enhancing plant physical defence against piercing-sucking insects. For example, a study has shown that K_2_SiO_2_ reduced the population fitness of green peach aphid *Myzus persicae* on *Zinnia elegans* [37]. This could be partly attributed to the deposition of silicon beneath the cuticle, forming a physical barrier in the cell wall to prevent the penetration by insects [4, 38].

The work reported here also showed that commercial fungicide treatments (with or without silicon) increased *T*. *urticae* infestation more than the application of silicon alone. It therefore suggested that there might be interactions between the use of fungicide and the presence of *T*. *urticae*. Research demonstrated that the fungicides fenhexamid and cyprodinil+fludioxinil reduced the mortality of *T*. *urticae* inoculated with a fungal pathogen *Neozygites floridana*, a natural enemy of *T*. *urticae* [39]. The fungicide cyprodinil+fludioxinil was also found to reduce sporulation of *N*. *floridana*, thus subsequently inhibiting establishment of *N*. *floridana* in *T*. *urticae* populations. The grower at Maltmas farm uses both the fungicides fenhexamid and cyprodinil+fludioxinil, as well as biocontrol agents such as *B*. *bassiana* and predatory mites such as *N*. *cucumeris* for disease and pest control. Although it is not clear whether these two fungicides would have similar inhibition effects on *B*. *bassiana* as they had on *N*. *floridana*, the greater number of *T*. *urticae* in the fungicide only treatment indicated that frequent use of some fungicide applications may inhibit the sporulation and establishment of some biocontrol agents, thereby reducing their efficacy against target pests.

It is considered that the location where silicon is deposited in the plant and how it is deposited significantly influence plant resilience. Work in 2012 and 2013 demonstrated that the plants from the silicon root treatment had less severe *P*. *aphanis* severity, and also a delayed epidemic development by up to two weeks, than those from the foliar treatment [11, 15]. Another study showed that strawberry plants were less susceptible to *P*. *aphanis* when grown in the silicon-saturated soil prior to planting than those treated with silicon after the first infection [33]. Therefore, root application of silicon was used in both 2014 and 2015 experiments. When silicon was applied through the fertigation system, it was then absorbed by plant roots, and transported through the xylem and finally deposited in the leaf epidermal cells and xylem vessels [8, 29]. Many other studies also reached similar conclusions; root silicon application stimulated plants both to form physical barriers and to interact with stress response metabolism, whereas foliar applied silicon may only create a chemical–physical barrier (e.g. a change in surface pH) through its deposition on the leaf surface [8, 29, 40]. Thus, good pest and disease management can be achieved by a continuous supply of silicon via the roots, resulting in improved plant resilience, and potentially reduce usage of pesticides [18], which subsequently contribute to sustainable management of strawberry production.

## Conclusions

Results from the two seasons of silicon fertigation experiments suggested that silicon improved strawberry plants’ resilience against strawberry powdery mildew and the infestation of two-spotted spider mites. Silicon was applied in a bioavailable form via the fertigation system and was taken up by the plants through the roots. It was suggested that the use of silicon could play an important role in the combined integration of pest and disease management in sustainable strawberry production.

## Supporting information

S1 Fig Strawberry production system in the 2014 and 2015 silicon fertigation experiments. (A) The strawberry polyethylene tunnel consisted of five growing beds (indicated by red arrows) in Pheasant Field, May 2015; (B) Strawberry plants were grown in 1m coir bags (i) on raised soil beds (ii), silicon nutrient plus water and fertilizers were fed to plants via irrigation drippers (iii).
(TIFF)

S2 Fig **Illustration of the (A) 2014 Blackberry Field (08 April–12 August) and (B) 2015 Pheasant Field (21 April-29 September) silicon fertigation experiments.** Each treatment block consisted of five growing beds each 15m long. Silicon nutrient was applied once per week at a concentration of 0.017% (by volume) through the fertigation tubes from 09 May in 2014 and from 22 April in 2015. Commercial fungicide was applied following the normal farm spray schedule.
(TIFF)

S1 Dataset 2014–2015 silicon fertigation experiments strawberry powdery mildew and two-spotted spider mites assessment results. (XLSX)

S2 Dataset 2014–2015 silicon fertigation experiments statistical analysis. Data on the severity of strawberry powdery mildew and two-spotted spider mite, and analysis of leaf silicon content was included.
(DOCX)
