# Supplementary figures and images for: Silicon builds resilience in strawberry plants against both strawberry powdery mildew Podosphaera aphanis and two-spotted spider mites Tetranychus urticae
Source: PLoS One. 2020 Dec 8;15(12):e0241151. doi: 10.1371/journal.pone.0241151 (PMC7723277; doi:10.1371/journal.pone.0241151)

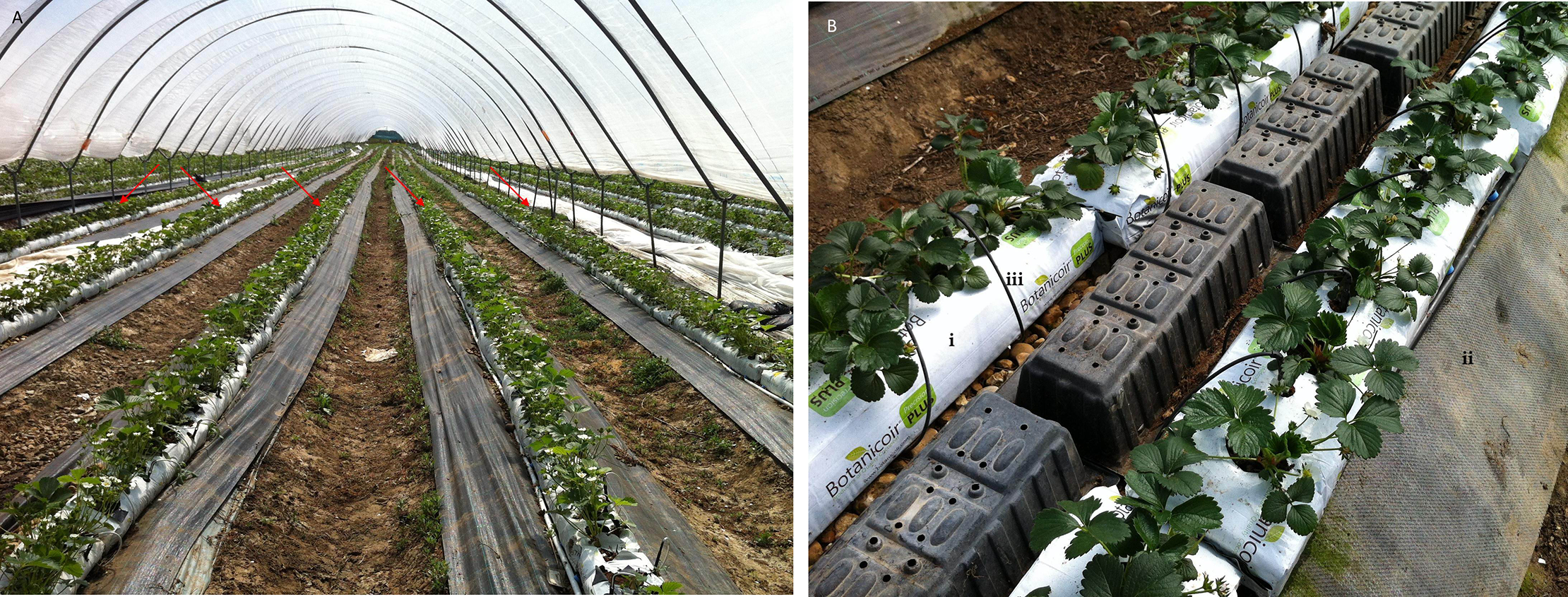

Supplement: S1 Fig — (A) The strawberry polyethylene tunnel consisted of five growing beds (indicated by red arrows) in Pheasant Field, May 2015; (B) Strawberry plants were grown in 1m coir bags (i) on raised soil beds (ii), silicon nutrient plus water and fertilizers were fed to plants via irrigation drippers (iii). (TIFF) [file pone.0241151.s001.tiff]

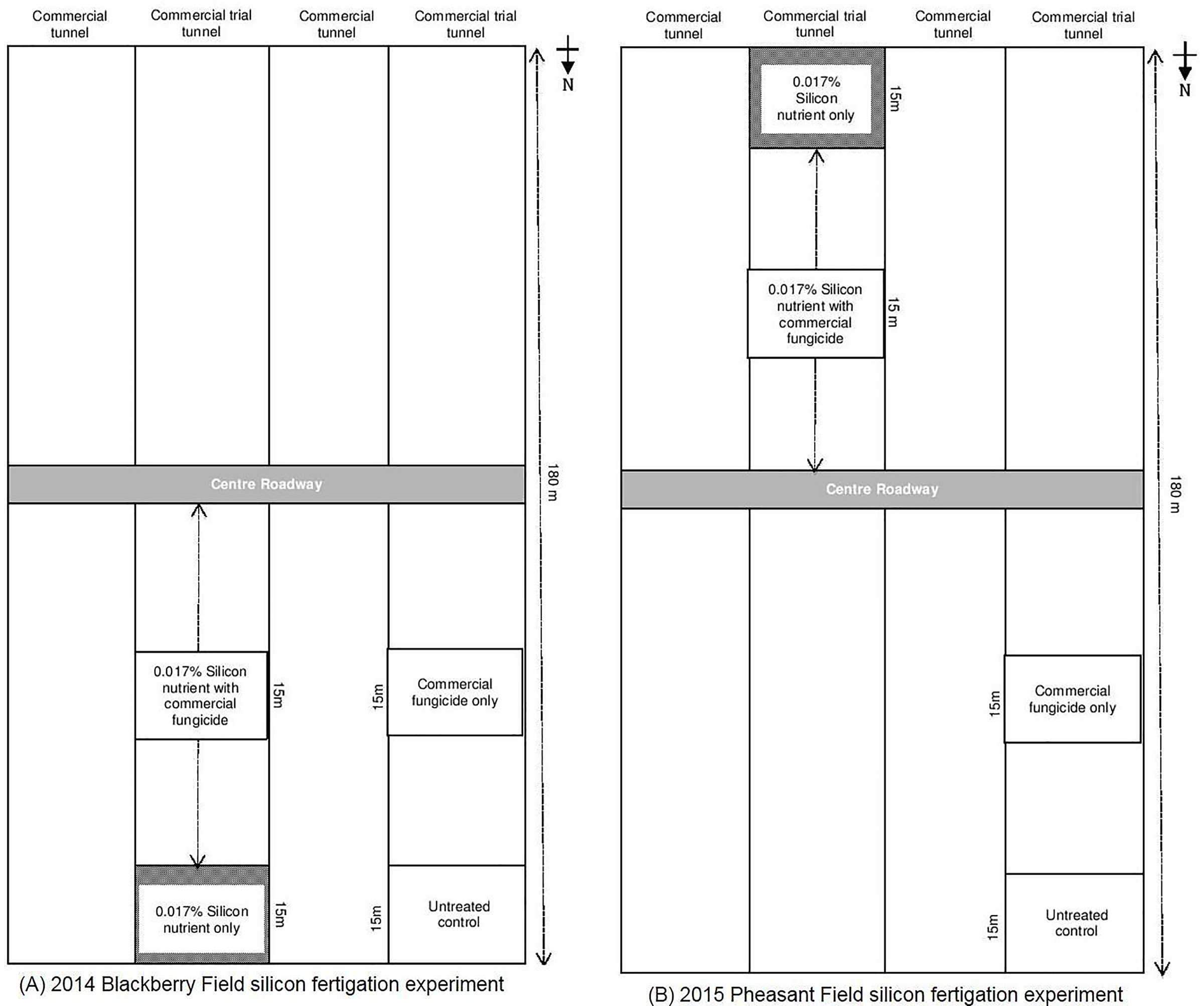

Supplement: S2 Fig — Illustration of the (A) 2014 Blackberry Field (08 April–12 August) and (B) 2015 Pheasant Field (21 April-29 September) silicon fertigation experiments. Each treatment block consisted of five growing beds each 15m long. Silicon nutrient was applied once per week at a concentration of 0.017% (by volume) through the fertigation tubes from 09 May in 2014 and from 22 April in 2015. Commercial fungicide was applied following the normal farm spray schedule. (TIFF) [file pone.0241151.s002.tiff]
