# Supplementary material for: Silicon builds resilience in strawberry plants against both strawberry powdery mildew Podosphaera aphanis and two-spotted spider mites Tetranychus urticae
Source: PLoS One. 2020 Dec 8;15(12):e0241151. doi: 10.1371/journal.pone.0241151 (PMC7723277; doi:10.1371/journal.pone.0241151)
Supplement: S2 Dataset — Data on the severity of strawberry powdery mildew and two-spotted spider mite, and analysis of leaf silicon content was included. (DOCX) [file pone.0241151.s004.docx]

1. **The overall analysis of 2014-2015 *P. aphanis* infection results**

**Table 1.1** **2014-2015 AUDPC from *P. aphanis* assessment.**

| **Treatment** | **Year** | **AUDPC^a^** | **Si** | **F** |
| --- | --- | --- | --- | --- |
| Untreated control | 2014 | 600.6 | N | N |
| Untreated control | 2014 | 608.6 | N | N |
| Untreated control | 2014 | 454.7 | N | N |
| Untreated control | 2014 | 760.1 | N | N |
| Untreated control | 2014 | 884 | N | N |
| Commercial fungicide only | 2014 | 78.8 | N | Y |
| Commercial fungicide only | 2014 | 139.2 | N | Y |
| Commercial fungicide only | 2014 | 78.5 | N | Y |
| Commercial fungicide only | 2014 | 132.7 | N | Y |
| Commercial fungicide only | 2014 | 142.2 | N | Y |
| 0.017% Si alone | 2014 | 342.4 | Y | N |
| 0.017% Si alone | 2014 | 374.5 | Y | N |
| 0.017% Si alone | 2014 | 495.4 | Y | N |
| 0.017% Si alone | 2014 | 486.9 | Y | N |
| 0.017% Si alone | 2014 | 651 | Y | N |
| 0.017% Si plus commercial fungicide | 2014 | 91.1 | Y | Y |
| 0.017% Si plus commercial fungicide | 2014 | 58.5 | Y | Y |
| 0.017% Si plus commercial fungicide | 2014 | 61 | Y | Y |
| 0.017% Si plus commercial fungicide | 2014 | 107.2 | Y | Y |
| 0.017% Si plus commercial fungicide | 2014 | 101.9 | Y | Y |
| Untreated control | 2015 | 56.84 | N | N |
| Untreated control | 2015 | 86.59 | N | N |
| Untreated control | 2015 | 82.18 | N | N |
| Untreated control | 2015 | 66.22 | N | N |
| Untreated control | 2015 | 72.8 | N | N |
| Commercial fungicide only | 2015 | 17.29 | N | Y |
| Commercial fungicide only | 2015 | 26.04 | N | Y |
| Commercial fungicide only | 2015 | 14.84 | N | Y |
| Commercial fungicide only | 2015 | 22.89 | N | Y |
| Commercial fungicide only | 2015 | 24.36 | N | Y |
| 0.017% Si alone | 2015 | 50.4 | Y | N |
| 0.017% Si alone | 2015 | 39.34 | Y | N |
| 0.017% Si alone | 2015 | 14 | Y | N |
| 0.017% Si alone | 2015 | 22.4 | Y | N |
| 0.017% Si alone | 2015 | 77.56 | Y | N |
| 0.017% Si plus commercial fungicide | 2015 | 26.04 | Y | Y |
| 0.017% Si plus commercial fungicide | 2015 | 2.94 | Y | Y |
| 0.017% Si plus commercial fungicide | 2015 | 15.82 | Y | Y |
| 0.017% Si plus commercial fungicide | 2015 | 13.02 | Y | Y |
| 0.017% Si plus commercial fungicide | 2015 | 22.54 | Y | Y |
| ^a^For the analysis of AUDPC and *P*-value between two silicon treatments (0.017% Si alone and 0.017% Si plus commercial fungicide) and two treatments without silicon (untreated control and commercial fungicide only), the calculation for the 2015 data included data from 05/05/2015 (no disease epidemic on 21/04/2015) to 25/08/2015, to ensure fair comparison i.e. the same number of sampling dates (nine samplings) during the similar sampling period (April/May- August) between 2014 and 2015 experiments. | | | | |

**Table 1.2 R analysis for 2014-2015 *P. aphanis* results.**

|  | Df | Sum Sq | Mean Sq | F value | Pr(>F) |  |
| --- | --- | --- | --- | --- | --- | --- |
| Si | 1 | 41957 | 41957 | 7.566 | 0.00971 | ** |
| F | 1 | 637474 | 637474 | 114.953 | 4.01e-12 | *** |
| Year | 1 | 868832 | 868832 | 156.673 | 7.16e-14 | *** |
| SI:F | 1 | 22183 | 22183 | 4.000 | 0.05404. | . |
| Si:Year | 1 | 21322 | 21322 | 3.845 | 0.05865. | . |
| F:Year | 1 | 458934 | 458934 | 82.758 | 2.18e-10 | *** |
| Si:F:Year | 1 | 11230 | 11230 | 2.025 | 0.16440 |  |
| Residuals | 32 | 177456 | 5545 |  |  |  |

Signif. codes: 0 ‘***’ 0.001 ‘**’ 0.01 ‘*’ 0.05 ‘.’ 0.1 ‘ ’ 1

**Table 1.3 R analysis for 2014 *P. aphanis* results between two silicon treatments (0.017% Si alone and 0.017% Si plus commercial fungicide) and two treatments without silicon (untreated control and commercial fungicide only).**

|  | Df | Sum Sq | Mean Sq | F value | Pr(>F) |  |
| --- | --- | --- | --- | --- | --- | --- |
| Si | 1 | 61550 | 61550 | 5.661 | 0.0301 | * |
| F | 1 | 1089091 | 1089091 | 100.168 | 2.71e-08 | *** |
| SI:F | 1 | 32490 | 32490 | 2.988 | 0.1031 |  |
| Residuals | 16 | 173963 | 10873 |  |  |  |

Signif. codes: 0 ‘***’ 0.001 ‘**’ 0.01 ‘*’ 0.05 ‘.’ 0.1 ‘ ’ 1

**Table 1.4 R analysis for 2015 *P. aphanis* results.**

|  | Df | Sum Sq | Mean Sq | F value | Pr(>F) |  |
| --- | --- | --- | --- | --- | --- | --- |
| Si | 1 | 1730 | 1730 | 7.922 | 0.0125 | * |
| F | 1 | 7317 | 7317 | 33.513 | 2.77e-05 | *** |
| SI:F | 1 | 923 | 923 | 4.228 | 0.0565 | . |
| Residuals | 16 | 3493 | 218 |  |  |  |

Signif. codes: 0 ‘***’ 0.001 ‘**’ 0.01 ‘*’ 0.05 ‘.’ 0.1 ‘ ’ 1

1. **The overall analysis of 2014-2015 *T. urticae* infestation results**

**Table 2.1** **2014-2015 AUPPC (overall number of *T. urticae* per treatment) from *T. urticae* assessment.**

| **Treatment** | **Year** | **SPM** | **Si** | **F** |
| --- | --- | --- | --- | --- |
| Untreated control | 2014 | 319 | N | N |
| Untreated control | 2014 | 417 | N | N |
| Untreated control | 2014 | 823 | N | N |
| Untreated control | 2014 | 582 | N | N |
| Untreated control | 2014 | 232 | N | N |
| Commercial fungicide only | 2014 | 1288 | N | Y |
| Commercial fungicide only | 2014 | 1144 | N | Y |
| Commercial fungicide only | 2014 | 1484 | N | Y |
| Commercial fungicide only | 2014 | 1826 | N | Y |
| Commercial fungicide only | 2014 | 1136 | N | Y |
| 0.017% Si alone | 2014 | 128 | Y | N |
| 0.017% Si alone | 2014 | 343 | Y | N |
| 0.017% Si alone | 2014 | 132 | Y | N |
| 0.017% Si alone | 2014 | 77 | Y | N |
| 0.017% Si alone | 2014 | 116 | Y | N |
| 0.017% Si plus commercial fungicide | 2014 | 59 | Y | Y |
| 0.017% Si plus commercial fungicide | 2014 | 198 | Y | Y |
| 0.017% Si plus commercial fungicide | 2014 | 126 | Y | Y |
| 0.017% Si plus commercial fungicide | 2014 | 162 | Y | Y |
| 0.017% Si plus commercial fungicide | 2014 | 163 | Y | Y |
| Untreated control | 2015 | 1664 | N | N |
| Untreated control | 2015 | 1167 | N | N |
| Untreated control | 2015 | 1387 | N | N |
| Untreated control | 2015 | 684 | N | N |
| Untreated control | 2015 | 229 | N | N |
| Commercial fungicide only | 2015 | 438 | N | Y |
| Commercial fungicide only | 2015 | 739 | N | Y |
| Commercial fungicide only | 2015 | 979 | N | Y |
| Commercial fungicide only | 2015 | 403 | N | Y |
| Commercial fungicide only | 2015 | 587 | N | Y |
| 0.017% Si alone | 2015 | 280 | Y | N |
| 0.017% Si alone | 2015 | 82 | Y | N |
| 0.017% Si alone | 2015 | 294 | Y | N |
| 0.017% Si alone | 2015 | 108 | Y | N |
| 0.017% Si alone | 2015 | 61 | Y | N |
| 0.017% Si plus commercial fungicide | 2015 | 223 | Y | Y |
| 0.017% Si plus commercial fungicide | 2015 | 73 | Y | Y |
| 0.017% Si plus commercial fungicide | 2015 | 379 | Y | Y |
| 0.017% Si plus commercial fungicide | 2015 | 280 | Y | Y |
| 0.017% Si plus commercial fungicide | 2015 | 121 | Y | Y |
| ^a^For the analysis of overall sum of *T. urticae* per treatment (i.e. AUPPC) and *P*-value between two silicon treatments (0.017% Si alone and 0.017% Si plus commercial fungicide) and two treatments without silicon (untreated control and commercial fungicide only), the calculation for the 2015 data included data from 19/05/2015 to 11/08/2015, to ensure fair comparison i.e. the same number of sampling dates (seven samplings) during the similar sampling period (mid May - August) between 2014 and 2015 experiments. | | | | |

**Table 2.2 R analysis for 2014-2015 *T. urticae* results.**

|  | Df | Sum Sq | Mean Sq | F value | Pr(>F) |  |
| --- | --- | --- | --- | --- | --- | --- |
| Si | 1 | 4986478 | 4986478 | 70.849 | 1.28e-09 | *** |
| F | 1 | 179962 | 179962 | 2.557 | 0.119639 |  |
| Year | 1 | 8323 | 8323 | 0.118 | 0.733181 |  |
| SI:F | 1 | 138886 | 138886 | 1.973 | 0.169728. |  |
| Si:Year | 1 | 46991 | 46991 | 0.668 | 0.419911 |  |
| F:Year | 1 | 945870 | 945870 | 13.439 | 0.000886 | *** |
| Si:F:Year | 1 | 1165881 | 1165881 | 16.565 | 0.000288 | *** |
| Residuals | 32 | 2252217 | 70382 |  |  |  |

Signif. codes: 0 ‘***’ 0.001 ‘**’ 0.01 ‘*’ 0.05 ‘.’ 0.1 ‘ ’ 1

**Table 2.3 R analysis for 2014 *T. urticae* results.**

|  | Df | Sum Sq | Mean Sq | F value | Pr(>F) |  |
| --- | --- | --- | --- | --- | --- | --- |
| Si | 1 | 3000800 | 3000800 | 78.98 | 1.38e-07 | *** |
| F | 1 | 975494 | 975494 | 25.68 | 0.000114 | *** |
| SI:F | 1 | 1054782 | 1054782 | 27.76 | 7.64e-05 | *** |
| Residuals | 16 | 607872 | 37992 |  |  |  |

Signif. codes: 0 ‘***’ 0.001 ‘**’ 0.01 ‘*’ 0.05 ‘.’ 0.1 ‘ ’ 1

**Table 2.4 R analysis for 2015 *T. urticae* results.**

|  | Df | Sum Sq | Mean Sq | F value | Pr(>F) |  |
| --- | --- | --- | --- | --- | --- | --- |
| Si | 1 | 2032669 | 2032669 | 19.779 | 0.000405 | *** |
| F | 1 | 150338 | 150338 | 1.463 | 0.244048 |  |
| SI:F | 1 | 249985 | 249985 | 2.432 | 0.138407 |  |
| Residuals | 16 | 1644344 | 102772 |  |  |  |

Signif. codes: 0 ‘***’ 0.001 ‘**’ 0.01 ‘*’ 0.05 ‘.’ 0.1 ‘ ’ 1

**3. 2014-2015 Leaf Si content analysis**

**3.1 2014 leaf Si content analysis**

> TukeyHSD(aov)

Tukey multiple comparisons of means

95% family-wise confidence level

Fit: aov(formula = Si ~ Treatment * Date, data = data1)

$Treatment

|  | diff | lwr | upr | P adj |
| --- | --- | --- | --- | --- |
| F-Control | 4.016648e+00 | -0.3850592 | 8.418356 | 0.0875204 |
| Si-Control | 7.124916e+00 | 2.7232087 | 11.526624 | 0.0002357 |
| SiF-Control | 7.124916e+00 | 2.7232087 | 11.526624 | 0.0002357 |
| Si-F | 3.108268e+00 | -1.2934397 | 7.509976 | 0.2628698 |
| SiF-F | 3.108268e+00 | -1.2934397 | 7.509976 | 0.2628698 |
| SiF-Si | -4.333494e-10 | -4.4017076 | 4.401708 | 1.0000000 |

Note^1^: Control – Untreated control; Si – 0.017% Si once a week, no commercial fungicides; F – Commercial fungicides only; SiF - 0.017% Si once a week, plus commercial fungicides.

**3.2 2015 leaf Si content analysis**

> TukeyHSD(aov)

Tukey multiple comparisons of means

95% family-wise confidence level

Fit: aov(formula = Si ~ Treatment * Date , data = data1)

$Treatment

|  | diff | lwr | upr | P adj |
| --- | --- | --- | --- | --- |
| F-Control | -0.32968519 | -4.43121457 | 3.77184420 | 1.0000000 |
| Si-Control | 0.81018519 | -3.29134420 | 4.91171457 | 0.9999640 |
| SiF-Control | 3.90668519 | -0.19484420 | 8.00821457 | 0.0788975 |
| Si-F | 1.13987037 | -2.96165901 | 5.24139975 | 0.9989972 |
| SiF-F | 4.23637037 | 0.13484099 | 8.33789975 | 0.0357667 |
| SiF-Si | 3.09650000 | -1.00502938 | 7.19802938 | 0.3563009 |

Note^1^: Control – Untreated control; Si – 0.017% Si once a week, no commercial fungicides; F – Commercial fungicides only; SiF - 0.017% Si once a week, plus commercial fungicides.
